# Using Environmental DNA to Estimate the Distribution of an Invasive Fish Species in Ponds

**DOI:** 10.1371/journal.pone.0056584

**Published:** 2013-02-20

**Authors:** Teruhiko Takahara, Toshifumi Minamoto, Hideyuki Doi

**Affiliations:** 1 Institute for Sustainable Sciences and Development, Hiroshima University, Higashi-Hiroshima, Japan; 2 Research Institute for Humanity and Nature, Kyoto, Japan; 3 Graduate School of Human Development and Environment, Kobe University, Kobe, Japan; Aberystwyth University, United Kingdom

## Abstract

Knowledge of the presence of an invasive species is critical to monitoring the sustainability of communities and ecosystems. Environmental DNA (eDNA), DNA fragments that are likely to be bound to organic matters in the water or in shed cells, has been used to monitor the presence of aquatic animals. Using an eDNA-based method, we estimated the presence of the invasive bluegill sunfish, *Lepomis macrochirus*, in 70 ponds located in seven locales on the Japanese mainland and on surrounding islands. We quantified the concentration of DNA copies in a 1 L water sample using quantitative real-time polymerase chain reaction (qPCR) with a primer/probe set. In addition, we visually observed the bluegill presence in the ponds from the shoreline. We detected bluegill eDNA in all the ponds where bluegills were observed visually and some where bluegills were not observed. Bluegills were also less prevalent on the islands than the mainland, likely owing to limited dispersal and introduction by humans. Our eDNA method simply and rapidly detects the presence of this invasive fish species with less disturbance to the environment during field surveys than traditional methods.

## Introduction

Biological invasions, often as a result of human action, are a serious threat to ecosystems throughout the world [1]–[4]. The introduction of invasive alien species often causes ecological disruptions at both the community and ecosystem levels. For example, the invasion of common carp (*Cyprinus carpio*) and red swamp crayfish (*Procambarus clarkii*) dramatically alters nutrient dynamics and/or the distribution of submerged macrophytes [5]. The distribution of the invasive species is often unknown and can change very rapidly. Given this, attempts should be made to monitor the expansions of their distributions [1], [4]. Early detection of invasive species is critical to limiting the dispersal and settlement of the invader [6].

Recently, there has been significant interest in developing methods for the detection of environmental DNA (eDNA) to allow the monitoring of species from DNA present in samples of freshwater [7]–[10] and seawater [11], [12]. Detection of short, species-specific DNA fragments in the water can increase the accuracy and decrease the cost of surveys and allow detection of target species [13], [14]. In the field of invasive species research, this approach was first used to detect the presence of the American bullfrog, *Rana catesbeiana* ( = *Lithobates catesbeianus*) in Western Europe [15]. The utility of eDNA detection has also been demonstrated in monitoring programs for bighead carp (*Hypophthalmichthys nobilis*) and silver carp (*H. molitrix*), two Asian carp species that have invaded much of North America’s Mississippi River [16]. Moreover, because eDNA based methods are relatively low impact to the environment, they offer the ability to investigate the distribution of invasive species in undisturbed environments that potentially contain rare species.

Bluegill sunfish, *Lepomis macrochirus*, is one of the most widely distributed species in Japanese freshwater ecosystems [17], [18] and is listed as an invasive species under the Invasive Alien Species Act of Japanese Law [19]. Although bluegill has spread to most areas in Japan following their initial introduction [17], [18], little is known about their presence in individual lakes/ponds.

Our objective was to develop the eDNA-based method to estimate the presence of invasive bluegill sunfish. In addition, we compared the presence of bluegill in the ponds on mainland or surrounding islands. We hypothesized that invasive bluegill would not be distributed as widely throughout the islands because there are no direct linkages between the ponds on the islands and mainland. We evaluated the distribution of bluegill in the ponds on the mainland and on islands in the Seto Inland Sea based on detection of eDNA and visual observation.

## Materials and Methods

### Study Species

The target species was bluegill sunfish, *L. macrochirus*. Comparisons of mitochondrial DNA between introduced populations in Japan and native populations in the USA indicate that the Japanese populations were derived from a small number of individuals from a population in the Mississippi River at Guttenberg, Iowa, USA [20]. Eighteen individuals from this population were imported into Japan in 1960. Based on official records, 15 of 18 individuals survived to leave offspring [21]. Some of these offspring were released or escaped into nearby lakes, including Lake Ippeki and Lake Biwa, while others were given to prefectural experimental stations and fishermen [22]–[24]. In the study region (Hiroshima Prefecture), bluegill invaded in the 1960’s [25], [26]. Human introductions have since resulted in the species expanding its distribution to ponds and lakes across Japan. As a result, bluegill is now dominant over native fishes in many lake habitats [27].

### Field Survey and DNA Extraction

**Figure 1:**
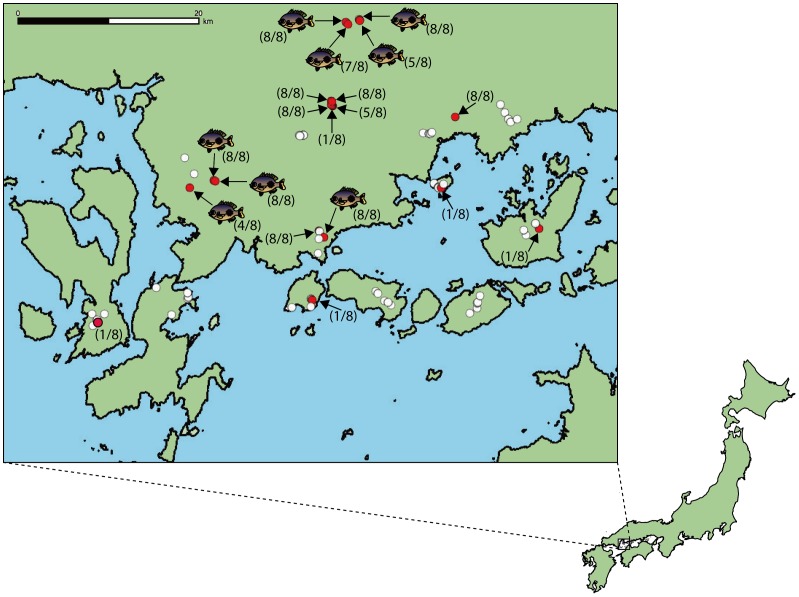
Estimated presence/non-detection of bluegill sunfish *Lepomis macrochirus* in the study area based on the detection of environmental DNA (eDNA) in the 70 study ponds. The red (n = 19) and white circles (n = 51) indicate the presence and non-detection of the bluegill eDNA, respectively. The fish illustrations (n = 8) show the ponds where bluegill was visually observed by the field survey. The ratios near the red circles/fish illustrations denote the number amplified out of eight replications in qPCR assay in each pond.

We surveyed the distribution of fish in 70 ponds that were located on seven islands and across seven mainland locales (34°05′–34°14′ N, 132°16′ –132°32′ E, see Fig. 1). We collected a 1 L water sample from the surface of each pond between 12∶00–16∶00 and during the period 19 October to 22 December 2011. In addition, we recorded the presence and non-detection of bluegill based on visual observations from the shore. A person observed the bluegill in the water with walking of the whole shoreline for 10–20 min depending on the shoreline length. No specific permits were required for the described field studies. The location is not privately-owned or protected in any way, and the field studies did not involve endangered or protected species.

We quantified eDNA using the method developed by Takahara et al. [28]. In brief, the water samples were stored in DNA-free 1 L bottles (Nalgene®) and immediately transported on ice in a cooling box to the laboratory, and stored at –30°C until the following procedure. After thawing, the water samples were filtered through a 3.0 µm membrane filter (cellulose acetate, 142 mm diameter, C300A142C; Advantec, Saijo, Japan) using stainless steel filter holders (KS-142-US; Advantec). This pore-size filter was found to be the most suitable for concentrating water samples in our previous study [28]. Each filter disc containing the sample was folded inward with tweezers and wrapped in DNA-free aluminum foil. The filter disc was immediately stored at –25°C until further analysis. All filtration equipment was carefully rinsed with distilled water between filtration operations to prevent cross-contamination.

To elute bluegill eDNA on the filter surface, the filter discs were placed in autoclaved 500-mL Nalgene® bottles using tweezers. The filter discs in each bottle were soaked in 10 mL autoclaved ultrapure water and stirred on a rotary shaker at maximum speed for 10 min. The suspension in the bottle was decanted into centrifugal filtration (Amicon Ultra-15, 30-kDa cutoff, UFC903096; Millipore, Billerica, MA, USA) and then concentrated by at 5000×g. The time required for centrifugation fluctuated between 10–20 min according to the differences of the clog conditions for each sample. These procedures were repeated three times for each filter disc. The sample solutions eluted from each disc were adjusted to volumes of 400 µL and stored in 1.5 mL microtubes (Eppendorf®) at –25°C. The eDNA from each whole sample solution (i.e. 400 µL) was extracted using a DNeasy blood and tissue kit (Qiagen, Hilden, Germany) in a final volume of 100 µL, with the following minor adjustments; 40 µL of Proteinase K and 400 µL of AL Buffer were added to each tube and then the tubes were incubated at 56°C for 60 min. After that, 400 µL of ethanol was added to each tube.

The six of 1-L autoclaved ultrapure water at –30°C were prepared as the blank of sample filtration and eDNA extraction. The single blank was filtered in the same manner after the filtration of samples in each working day. After that, all blanks were treated as equals of the samples for eDNA extraction and qPCR procedure, and resulted non-detected bluegill eDNA in subsequent qPCR assay.

### Real-time Quantitative PCR

The quantification of eDNA was performed using real-time TaqMan® PCR with a StepOne-Plus™ Real-Time PCR system (Life Technologies, City of Carlsbad, CA, USA). The mitochondrial cytochrome *b* gene fragments were amplified and quantified with the following primers: Bluegill_CytB_F (5′- GCCTAGCAACCCAGATTTTAACA-3′), Bluegill_CytB_R (5′- ACGTCCCGGCAGATGTGT-3′), and Bluegill_CytB_probe (5′-FAM- CGACATCGCAACTGCCTTCTCTTCAGT-TAMRA-3′). These primers were specific to bluegill and amplify a 100 bp fragment of the cytochrome *b* gene. The specificity of the primers was tested with the sequences of all sunfish species that are present in Japan (i.e., *Micropterus salmoides*, *M. dolomieu* and *L. macrochirus*). Non-target species which potentially inhabited the study sites were not detected during the *in silico* specificity screen, which was performed using Primer-BLAST with default settings (http://www.ncbi.nlm.nih.gov/tools/primer-blast/).

Each TaqMan reaction contained 900 nM of each primer and 125 nM TaqMan probe in 1× PCR master mix (TaqMan gene expression master mix; Life Technologies) and 2 µL of the DNA solution. This volume of eDNA solution treated in this study was found to be appropriate for qPCR procedure according to our preliminary analysis (Takahara et al. unpublished data), although it would be required to check the optimal volume of eDNA solution without PCR inhibition in each case. The total volume of each reaction mixture was 20 µL. The PCR conditions were as follows: 2 min at 50°C, 10 min at 95°C, and 55 cycles of 15 s at 95°C and 60 s at 60°C. Quantitative real-time PCR (qPCR) was performed in eight replications. PCR products of the target sequences were cloned into pGEM-T Easy Vector (Promega, Tokyo, Japan), and a dilution series of the plasmid containing 3×10^1^ to 3×10^4^ copies were amplified as standards in duplicate in all qPCR assays. Because one copy of the DNA was detected in at least one well in each replication, we defined the limit of detection (LOD) for bluegill DNA using qPCR assay as 1 copy. If any of eight replications for each pond yielded a positive result, it was assigned the presence of bluegill eDNA in the pond. Each qPCR assay included eight wells that contained no template to serve as a negative control. In all cases there was no amplification from these wells. Moreover, we used these primers in qPCR assay to amplify DNA extracted from the tissue of two closely related non-target species (*M. salmoides* and *M. dolomieu*). These tests resulted in no amplification. To avoid contamination, we performed PCR set-up, including preparation/addition of the standards, and qPCR cycling in two separate rooms.

### DNA Sequencing

To confirm the specificity of the primer set described above for the field samples, qPCR amplicons with probes and ROX in all sites that were positive for the qPCR were directly sequenced after treatment with ExoSAP-IT (USB Corporation, Cleveland, OH, USA). Sequences were determined by a commercial sequencing service (Takara Bio, Tokyo, Japan).

### Statistical Analyses

We used Fisher’s exact test to compare the proportion of ponds in which bluegill was detected based on visual observation or the presence of eDNA. We also tested for differences in the proportion of ponds that were occupied by bluegill on the mainland and islands using Fisher's exact test. The significances of all statistics were set as α = 0.05. All statistical analyses were conducted using R ver. 2.15.0 [29].

## Results

We detected the eDNA of bluegill sunfish in 19 of 70 ponds. Based on our results we plotted the distribution of the bluegill in our study area (Fig. 1). We detected eDNA in all the ponds where bluegills were directly observed (n = 8) and in some of the ponds where bluegills were not observed visually (11 of 62) (Fisher’s exact test, *p*<0.00001, n = 70, Table 1). Bluegills were distributed primarily on the mainland, except in one of five ponds on each of four islands. Thus, there was a significant difference in the proportion of ponds occupied by bluegill between the mainland and outlying islands (Fisher’s exact test, *p* = 0.0066, Table 2).

**Table 1:** Presence and non-detection (or not-observed) of bluegill sunfish, *Lepomis macrochirus*, in the ponds based on detection of environmental DNA (eDNA) and visual observation from shore.

		eDNA	
		Presence	Non-detection
Observation	Presence	8	0
	Not observed	11	51

The difference in proportion is significant among the combinations in the table (Fisher’s exact test, *p*<0.0001).

**Table 2:** Presence and non-detection of bluegill sunfish, *Lepomis macrochirus*, in ponds on the mainland or on surrounding islands based on the detection of environmental DNA (eDNA).

	Mainland	Islands
Presence	15	4
Non-detection	20	31

The difference in proportion is significant between the ponds on the mainland and islands (Fisher’s exact test, *p* = 0.0066).

To confirm the specificity of the primer set, we directly sequenced the qPCR amplicons. All sequences from each qPCR amplicon at ponds were confirmed as being from bluegill, *L. macrochirus*.

## Discussion

We developed a method for determining the presence of an invasive fish species, *L. macrochirus*, based on the detection of eDNA in water samples. We were able to detect bluegill eDNA in all areas where the species was visually observed. Based on direct sequencing, we correctly detected DNA fragments from bluegill in the environmental samples. Thus, our method can be used to accurately detect the presence of bluegill.

Interestingly, we also detected bluegill eDNA in several ponds in which bluegills were not visually observed. The probability of detection based on observations from shore is influenced by a range of factors, including fish size, fish behavior, and physical habitats [30]. In general, these factors lead to underestimating the number of ponds occupied by bluegill. Given this, our method offers an alternative means of detecting presence with increased accuracy, particularly in small water-bodies such as ponds, where the water is well mixed [31]. In larger lakes and reservoirs, detection probability based on eDNA will be influenced by the number of fish present in a given area. For example, Takahara et al. (2012) found large differences in eDNA concentrations from common carp among the sites in a large lagoon [28].

Absence of species is difficult to confirm by sampling and/or observation [30]. Estimation of the potential absence of species is a significant issue for programs that monitor populations and predict their distribution [32]. Though we were able to detect at least one copy of DNA using our current protocol, we only collected 1 L of pond surface water for analysis. Therefore, we cannot confirm the “absence” of the target species from our analysis. Current eDNA methods, including ours, are focused primarily on detecting species presence, but not absence. However, there is a definite need to develop methods to detect the “presence/absence” of a species.

In summary, we developed a method for estimating the distribution of an invasive fish species based on the detection of eDNA. Using this method, the presence of invasive species can be estimated more precisely than using traditional methods, such as casting-nets or fishing in the natural environments where species are difficult to detect. This method can be easily adapted to monitor other invasive species for which primers are available or can be developed.

We confirmed our hypothesis that bluegills are more prevalent in ponds on the mainland than on surrounding islands. This pattern may be explained by a number of factors, including 1) limitation of dispersal without direct water linkages between the ponds on the islands and between the islands and mainland, and 2) limitation of introductions by humans. Indeed, the human population is relatively low on all seven islands (100 to 27,000 people: e-Stat, Japanese National Statistics Center, http://www.e-stat.go.jp/[in Japanese]). However, our results suggest that despite this isolation one of five ponds on each of four islands was already invaded by bluegill, indicating the beginning of dispersal in the islands. In general, islands have many endemic and rare species [33], [34], so they offer particularly high conservation value and warrant a high priority for biodiversity conservation [34]. Currently, bluegills have not dispersed throughout the islands, but we advocate monitoring their distribution more closely to conserve the unique aquatic communities on these islands.
